# Seed Priming with Fullerol Improves Seed Germination, Seedling Growth and Antioxidant Enzyme System of Two Winter Wheat Cultivars under Drought Stress

**DOI:** 10.3390/plants12061417

**Published:** 2023-03-22

**Authors:** Haiyan Kong, Xiangzhan Meng, Nudrat Aisha Akram, Fengru Zhu, Jiaxing Hu, Zhen Zhang

**Affiliations:** 1 School of Resources and Environment, Anhui Agricultural University, Hefei 230036, China; 2 Department of Botany, Government College University, Faisalabad 38040, Pakistan

**Keywords:** fullerol, seedling growth, antioxidant system, winter wheat, drought stress

## Abstract

The application of carbon-based nanomaterials (CBNMs) in plant science and agriculture is a very recent development. Many studies have been conducted to understand the interactions between CBNMs and plant responses, but how fullerol regulates wheat subjected to drought stress is still unclear. In this study, seeds of two wheat cultivars (CW131 and BM1) were pre-treated with different concentrations of fullerol to investigate seed germination and drought tolerance. Our results indicate that the application of fullerol at certain concentrations (25–200 mg L^−1^) significantly promoted seed germination in two wheat cultivars under drought stress; the most significant effective concentration was 50 mg L^−1^, which increased the final germination percentage by 13.7% and 9.7% compared to drought stress alone, respectively. Wheat plants exposed to drought stress induced a significant decrease in plant height and root growth, while reactive oxygen species (ROS) and malondialdehyde (MDA) contents increased significantly. Interestingly, wheat seedlings of both cultivars grown from 50 and 100 mg L^−1^ fullerol-treated seeds were promoted in seedling growth under water stress, which was associated with lower ROS and MDA contents, as well as higher antioxidant enzyme activities. In addition, modern cultivars (CW131) had better drought adaptation than old cultivars (BM1) did, while the effect of fullerol on wheat had no significant difference between the two cultivars. The study demonstrated the possibility of improving seed germination, seedling growth and antioxidant enzyme activities by using appropriate concentrations of fullerol under drought stress. The results are significant for understanding the application of fullerol in agriculture under stressful conditions.

## 1. Introduction

Wheat (*Triticum aestivum* L.) is one of the major cereal crops, feeding more than half of the world’s population [1]. Drought is considered to be one of the most adverse abiotic stresses affecting plant growth and crop yield [2]. Drought stress leads to the production of reactive oxygen species (ROS), including hydrogen peroxide (H_2_O_2_) and a hydroxyl radical (·OH), which can cause damage to membrane lipid peroxidation [3]. Plants have developed an enzymatic antioxidant system, including total superoxide dismutase (SOD), catalase (CAT), and peroxidase (POD), for scavenging ROS in order to maintain individual growth and grain production, which is a ubiquitous mechanism for plants to cope with various abiotic stresses [4,5].

Carbon-based nanomaterials (CBNMs) are widely applied in agriculture, such as in plant growth and development regulation, soil fertility improvement, pest control, and environmental pollution elimination [6,7]. Among various CBNMs, carbon nanotubes, fullerene (C_60_) and its water-soluble derivatives, such as fullerol or fullerenol, are the most investigated CBNMs [8]. In recent years, a series of studies have been carried out to explore how fullerene and its derivatives regulate plant growth and development, some of which have shown beneficial effects on plants, while other reports have been negative or have indicated no effects [6]. Kole et al. [9] found that seed pre-treatment with fullerol had beneficial effects on biomass yield, water content, and fruit yield in bitter melon. Moreover, fullerol exposure (ranging from 10 to 100 mg L^−1^) significantly promoted seed germination, above-ground dry weight, and the photosynthesis of *B. napus* under water stress [10]. Fullerol application boosted early seedling growth and establishment in salinity-induced wheat [11]. Furthermore, fullerol added to the nutrient medium accelerated barley root elongation under stressful conditions [12].

The results also indicate that the application of fullerol can effectively improve the tolerance of plants under stress conditions. Foliar spraying of fullerol reduced drought-induced oxidative stress of sugar beets [13]. It has been speculated that fullerol can be used as a free radical scavenger or as an intracellular water binder under stress conditions, so that plants can adapt to drought stress [12,13]. Furthermore, foliar spraying of fullerol improved drought tolerance in canola seedlings by promoting the ability of antioxidant systems to reduce the levels of reactive oxygen species (ROS) [10]. Similarly, spraying exogenous fullerol or using it in seed priming both improved antioxidant defense, reducing H_2_O_2_ content in wheat under salt stress [11,14].

Therefore, although significant efforts have been made to understand the interactions between fullerol and plant responses, the influence of fullerol on wheat exposed to drought stress is not well understood. Therefore, the objective of this study was to evaluate the role of fullerol on seed germination, seedling growth, and alleviating oxidative stress in two winter wheat cultivars when plants were exposed to drought stress stimulated by polyethylene glycol (PEG).

## 2. Results

### 2.1. Trial 1: Effect of Fullerol on Seed Germination in Two Winter Wheat Cultivars

#### 2.1.1. Characterization of Fullerol Nanoparticles

**Figure 1:**
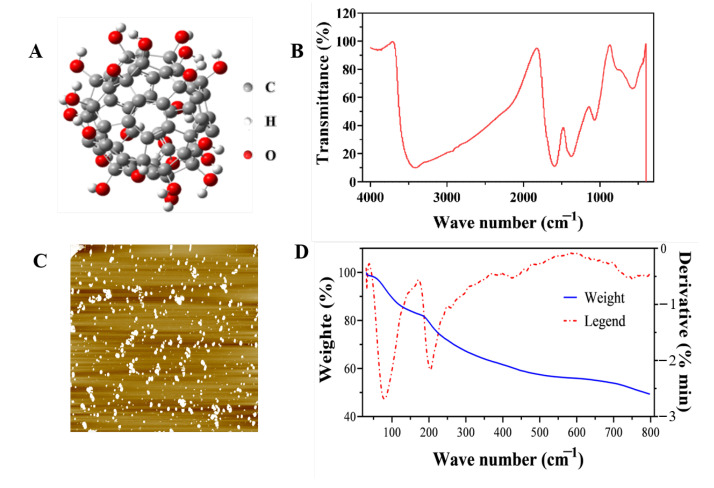
Structure and characterization of fullerol. (**A**) Molecular structure diagram of fulerol; (**B**) infrared spectra; (**C**) atomic force microscope; (**D**) thermogravimetric and differential thermal analysis (TG-GTA).

Four peaks centered at 3415 cm^−1^, 1953 cm^−1^, 1378 cm^−1^ and 1073 cm^−1^ were measured for C60 in a KBr pellet (Figure 1B). The stretching vibration peak of C-O-C and C-O are 1953 cm^−1^ and 1073 cm^−1^, and 3415 cm^−1^ and 1378 cm^−1^ are the stretching vibration peaks of O-H and C-O-H, respectively (Figure 1B). AFM images showed that fullerol nanoparticles had good dispersion and uniform morphology (Figure 1C). Thermogravimetric and differential thermal analysis (TG-GTA) (Figure 1D and Figure S1) and elemental analysis (Table S1) were used to measure the thermal stability, crystal water, and hydroxyl groups of fullerol nanoparticles. From TG-GTA, fullerol contains 16.03% H_2_O, and the contents of H and C are 2.453% and 37.94%, as shown by elemental analysis. It can be concluded, via calculation, that the sample contained about 12% H_2_O and 23% OH^−^. The molecular formula of the sample is C60(OH)_23_.12H_2_O (Figure 1D). Therefore, it is suggested that the fullerol nanoparticles used in this experiment were monodisperse and stable for better uptake by wheat plants.

#### 2.1.2. Seed Germination Parameters

Drought stress stimulated by PEG treatment resulted in a significant decrease in seed germination of CW131 and BM1 (Table 1). Interestingly, seeds exposed to fullerol at appropriate concentrations significantly reversed the inhibition of PEG on seed germination in two wheat cultivars (Table 1). Fullerol concentrations between 25–200 mg L^−1^ significantly promoted the final germination percentage, germination index, and seed vigor index in CW131 under drought stress; the most significant effective concentration was 50 mg L^−1^, which increased these three indices by 13.7%, 36.5%, and 54.2% compared to drought stress alone, respectively (Table 1). Under the drought stress condition, the radical plus plumule length of CW131 treated with 25–100 mg L^−1^ of fullerol was enhanced by 18.9%, 34.0%, and 17.0%, respectively, compared to the PEG addition alone (Table 1). Similarly, the addition of 25–100 mg L^−1^ of fullerol improved the seed final percentage and germination index, and the 25–200 mg L^−1^ fullerol treatment induced the radical plus plumule length and seed vigor index in BM1 under drought stress; the most effective significant effective concentration was 50 mg L^−1^ (Table 1). In contrast, the high-concentration fullerol treatment (300 mg L^−1^) did not significantly promote seed germination in both cultivars under drought stress (Table 1). The values of seed germination in BM1 were significantly lower than those in CW131 (*p* < 0.05) (Table 1).

### 2.2. Trial 2: Effect of Fullerol on Drought Tolerance in Two Winter Wheat Cultivars

#### 2.2.1. Plant Height and Root Growth Parameters

**Figure 2:**
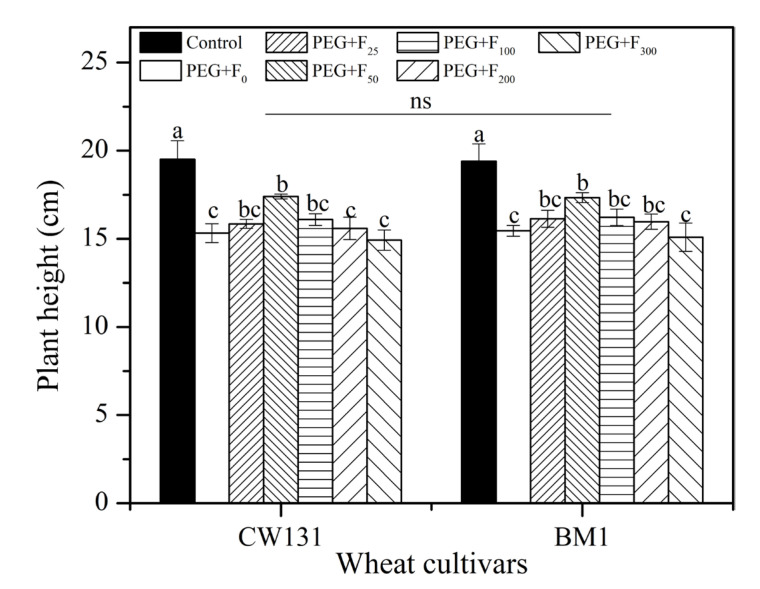
Effect of different fullerol additions (concentrations range from 0 to 300 mg L^−1^) on the plant heights of two wheat cultivars (CW131 and BM1) under PEG (20% polyethylene glycol) treatment. Mean values ± SE are shown (*n* = 3). Bars with different letters for the same cultivar imply a significant difference at *p* < 0.05. For ANOVA results, ns, not significant.

PEG treatment significantly reduced the plant heights of both wheat cultivars (Figure 2). The addition of 50 mg L^−1^ of fullerol increased plant heights in CW131 and BM1 by 13.7% and 12.3%, respectively, compared to the PEG treatment alone, while other concentrations had no significant effect on both cultivars (Figure 2). There was no significant difference in plant heights between CW131 and BM1 (*p* > 0.05).

PEG treatment also significantly reduced root length, root surface, and root volume, but had no significant impact on root mean diameter in both wheat cultivars (Table 2). The PEG-induced decreases in root growth of the two wheat cultivars were remarkably reversed by the fullerol treatment at 50 and 100 mg L^−1^. In contrast, other concentrations did not have the same effect (Table 2). The application of 50 mg L^−1^ of fullerol promoted root length, root surface, and root volume in CW131 by 18.3%, 26.8%, and 27.8%, respectively, compared to the PEG addition alone (Table 2). The threads of influence of fullerol on the root growth of BM1 were consistent with those on the root growth of CW131, and the values were significantly lower than those of CW131 (*p* < 0.05) (Table 2). There was no significant effect on root mean diameter in both wheat cultivars at any concentration of fullerol (*p* > 0.05). The effect of fullerol on root length, root surface, root volume and root mean diameter showed no significant differences between the two wheat cultivars (*p* > 0.05).

#### 2.2.2. ROS Level and Membrane Lipid Peroxidation (MDA)

**Figure 3:**
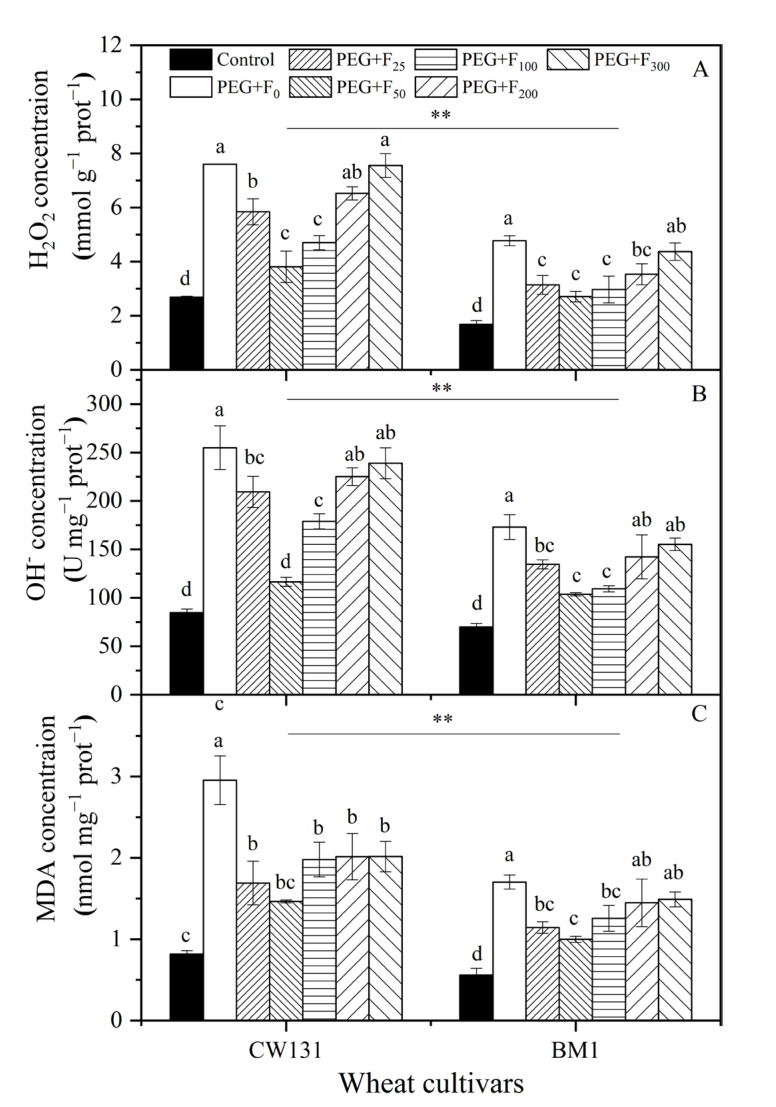
Changes in contents of hydrogen peroxide (H_2_O_2_) (**A**), hydroxyl radical (OH) (**B**), and malondialdehyde (MDA) (**C**) of two wheat cultivars (CW131 and BM1) under different fullerol additions (concentrations range from 0 to 300 mg L^−1^) and PEG (20% polyethylene glycol) treatment. Mean values ± SE are shown (*n* = 3). Bars with different letters for the same cultivar imply a significant difference at *p* < 0.05. For ANOVA results, **, *p* < 0.01.

PEG treatment significantly increased the generation of H_2_O_2_, ·OH, and MDA in the leaves of the two wheat plants, and the levels were higher in CW131 than in BM1 (Figure 3). Fullerol treatment significantly reduced the H_2_O_2_ level in CW131, especially at 25, 50, 100, and 200 mg L^−1^, which decreased by 34.2%, 43.3%, 37.8%, and 26.0%, compared to that under the PEG treatment alone, respectively (Figure 3A). The trends of the ·OH and MDA concentrations of the CW131 cultivar were consistent with the changes in H_2_O_2_ level. The level of ·OH in CW131 treated with 25, 50, and 100 mg L^−1^ of fullerol decreased by 22.2%, 40.1%, and 36.8%, respectively, compared to the PEG treatment alone (Figure 3B). Moreover, the fullerol treatment (25–100 mg L^−1^) reduced the accumulation of MDA in CW131, which decreased by 32.8%, 41.3%, and 26.2%, compared to the PEG treatment alone (Figure 3C). The impacts of different concentrations of fullerol on the contents of H_2_O_2_, ·OH, and MDA in BM1 were consistent with those in CW131, but the values were significantly lower than those in CW131 (*p* < 0.05) (Figure 3). The results indicate that fullerol treatment could reduce ROS levels and alleviate the damage of membrane lipid peroxidation under drought stress.

In addition, we fitted quadratic polynomial regression for the contents of H_2_O_2_, ·OH and MDA in two winter cultivars (CW131 and BM1) plotted against six concentrations of fullerol (0, 20, 50, 100, 200, and 300 mg L^−1^) and found that most of the fits were not very good (Figure S2). We further performed a lack-of-fit test (Tables S2–S4) and found that the line and quadratic models may not be good choices, while one-way ANOVA may be better supported, which we believe may be related to the low number of replicates.

#### 2.2.3. Antioxidant Enzyme Activities

**Figure 4:**
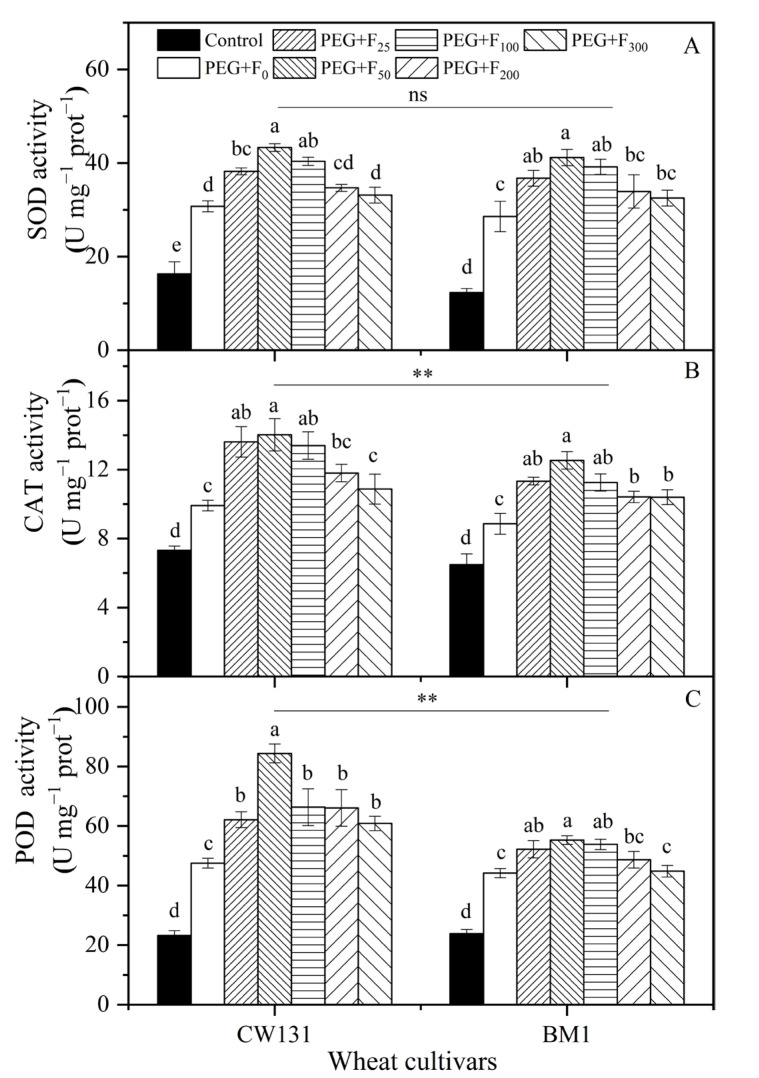
Activities of antioxidant enzymes: superoxide dismutase (SOD) (**A**), catalase (CAT) (**B**), and peroxidase (POD) (**C**) of two winter cultivars (CW131 and BM1) under different fullerol additions (concentrations range from 0 to 300 mg L^−1^) and PEG (20% polyethylene glycol) treatment. Mean values ± SE are shown (*n* = 3). Bars with different letters for the same cultivar imply a significantly difference at *p* < 0.05. For ANOVA results, **, *p* < 0.01; ns, not significant.

The PEG treatment triggered remarkable increases in the activities of SOD, CAT, and POD in the two wheat cultivars, and the levels were higher in CW131 than in BM1 (Figure 4). The fullerol concentrations between 25–100 mg L^−1^ increased the SOD, CAT, and POD activities under water stress both in CW131 and BM1; the most significant effective concentration was 50 mg L^−1^, while high concentrations (200 and 300 mg L^−1^) had no significant effect (Figure 4). The application of 50 mg L^−1^ of fullerol enhanced SOD activity in CW131 and BM1 by 40.9% and 44.1%, respectively, compared to PEG treatment alone (Figure 4A). Similarly, the CAT activity of the CW131 and BM1 wheat cultivars increased by 41.3% and 41.5% at the concentration of 50 mg L^−1^ of fullerol, respectively (Figure 4B). Moreover, the addition of fullerol had a similar promotional effect on the POD activity of CW131 and BM1, which was increased by 77.4% and 25.1% under the application of 50 mg L^−1^ of fullerol, respectively, compared to the PEG-only treatment. (Figure 4C).

Similarly, we fitted quadratic polynomial regression for the activities of SOD, CAT and POD in two winter cultivars (CW131 and BM1) plotted against six concentrations of fullerol (0, 20, 50, 100, 200, and 300 mg L^−1^) (Figure S2) and found the same results (Tables S4–S7).

## 3. Discussion

**Figure 5:**
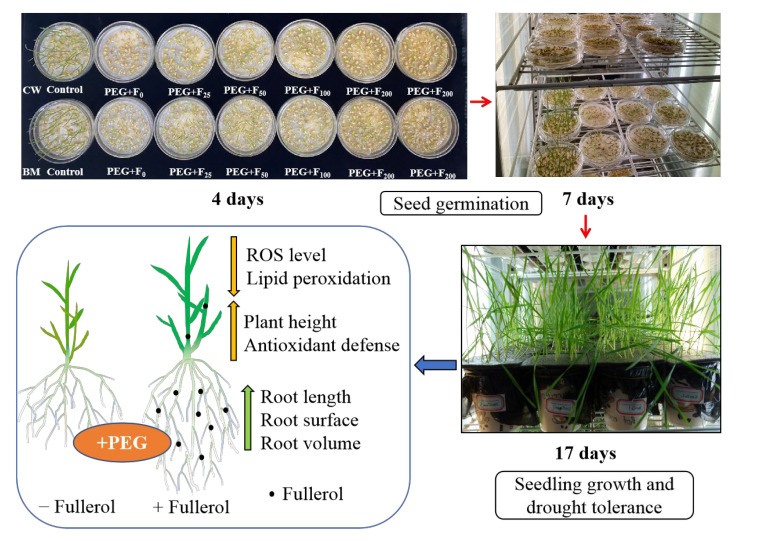
Model figure displaying the impact of fullerol on seed germination, as well as seedling growth and the antioxidant system in wheat subjected to drought stress. The seed germination experiment lasting 7 days demonstrated that seed priming with fullerol exhibited a positive effect on seed germination in wheat under PEG (polyethylene glycol) treatment. Moreover, it is suggested that wheat seedlings grown from fullerol-treated seeds were promoted in plant height and root growth under water stress, and this promotion was associated with the contents of lower reactive oxygen species (ROS) and malondialdehyde (MDA), as well as higher antioxidant activities of superoxide dismutase (SOD), catalase (CAT) and peroxidase (POD).

The present study revealed that fullerol regulated seed germination, seedling growth, and antioxidant systems in drought-induced wheat (Figure 5). The results demonstrate that fullerol administered in appropriate concentrations improved seed germination, plant height, and root growth, as well as increased antioxidant enzyme activities to reduce the accumulation of ROS and MDA in two wheat cultivars exposed to drought stress. The results in the present study open up the possibility of future applications of fullerol in agriculture under stress conditions.

Fullerol is an important water-soluble derivative of fullerene carbon-based nanomaterials (CBNMs) with unique properties, and has been increasingly used in agriculture, horticulture and biotechnology [15,16]. Many efforts have been made to understand the interaction between CBNMs and plant responses, but only a few studies have evaluated the use of fullerol in wheat under drought stress. The present study investigated seeds exposed to different concentrations of fullerol and their effects on seed germination and drought tolerance in two wheat cultivars (CW131 and BM1) under drought stress stimulated by 20% PEG.

PEG treatment significantly inhibited the seed germination of two wheat cultivars in this study, which is consistent with the findings of previous studies [17]. The results further showed that a low-concentration fullerol (25–200 mg L^−1^) treatment increased the seed’s final germination percentage, germination index, radicle plus plumule length, and seed vigor index in two wheat cultivars under water stress; the most significant effective concentration was 50 mg L^−1^. The results were supported by those of Liu et al. [18], who reported that the application of 50 mg L^−1^ of fullerol significantly promoted the seed germination rate, germination energy, and germination index in maize. This was also in accordance with previous studies showing that the promotion of seed germination in canola was more pronounced with the application of 10 and 100 mg L^−1^ of fullerol under water stress [10]. However, high concentrations of fullerol had no significant effect on wheat, which may be related to the inhibition of crop uptake at high concentrations. Previous work has indicated that wheat seedlings treated with relatively low concentrations of ^13^C-fullerenol nanoparticles showed significant increases in ^13^C content in the roots, while high concentrations of fullerenol appeared to inhibit this accumulation [19]. Much work has been done to study the mechanisms of fullerol affecting plants, and it is generally accepted that CBNMs have high mobility in plants tissues, especially in terms of their ability to penetrate the plant seed coats and regulate seed germination and plant growth [20,21]. Kole et al. [9] found that the accumulation of fullerol in the tissues and cells of the roots, stems, petioles, leaves, flowers, and fruit of bitter melon after the treatment of seeds with fullerol was related to the increased biomass yield and fruit yield of bitter melon. Borisev et al. [13] indirectly revealed that fullerol could penetrate the leaf and root tissues of sugar beets, which bind water in different cell compartments. The bioaccumulation of fullerol nanoparticles in wheat was directly confirmed using ^13^C-labeling technology, and could be transported from the roots to the stems and leaves [19]. Similarly, C60 fullerene has been shown to be absorbed by roots, and then transferred to the stems and panicles of rice [22]. According to the characterization data of fullerol nanoparticles, it is suggested that the fullerol nanoparticles used in this experiment were monodisperse and stable, and they were reported to be better absorbed by plants [23]. Similarly to these findings, we suggest that fullerol can be accumulated in wheat leaves after uptake by the root system. Therefore, we speculate that fullerol is highly mobile in plant tissues and can penetrate different biological membranes to regulate plant growth and adaptation.

In general, the growth process is inhibited when plants are subjected to water stress [24]. In agreement with this, we revealed that drought stress significantly reduced plant height and root growth in two wheat cultivars. Our work further indicated that pre-treatment with 50 mg L^−1^ of fullerol improved the plant heights of two wheat cultivars under drought stress, while other concentration treatments had no significant effect. Furthermore, fullerol treatment (50 and 100 mg L^−1^) significantly increased root growth under drought stress, including root length, root surface, and root volume. The result was consistent with the results of previous reports that indicated that fullerol nanoparticles significantly promoted the root and shoot growth in salt-stressed wheat at appropriate concentrations. [25]. Similarly, the beneficial effects of fullerol on plant growth have been reported for *Arabidopsis* [26], bitter melon [9], barley [12], and cucumber [15]. It is generally assumed that CBNMs promote seed germination and plant growth mainly because of their ability to facilitate water acquisition [27,28]. Exogenous nanoparticles improved plant growth by promoting water use efficiency, nutrient uptake, and accelerating root growth [29,30]. Moreover, it has been assumed that fullerol can act as an intracellular water binder, allowing plants to adapt to the effects of drought [13]. The results indicate that fullerol has great potential as a plant growth regulator in agricultural applications.

Drought-induced disturbances to ROS metabolism in plants were evidenced by the production of higher H_2_O_2_ and ·OH anion production, which also led to the accumulation of MDA, a by-product of cell membrane damage [31,32]. In this study, higher ROS generation and lipid peroxidation were detected in both drought-stressed wheat plants, and the modern cultivar, CW131, had lower ROS levels than the old cultivar, BM1, did. The same results were found in three winter cultivars [33] and two spring wheat cultivars [34]. Importantly, fullerol treatment (25–100 mg L^−1^) resulted in lower ROS and MDA levels, which alleviated drought damage to the wheat seedlings; the most significant concentration was 50 mg L^−1^. This observation is consistent with the idea that the foliar spraying of fullerol significantly reduced the leaf ROS and MDA contents of canola seedlings treated with certain concentrations of fullerol (1–100 mg L^−1^) under drought stress [10]. In agreement with this, the exogenous application of fullerol in seed priming and foliar spaying substantially improved the salinity tolerance of wheat by lowering H_2_O_2_ levels [11,14,25]. Fullerol has been reported to exhibit significant free radical scavenging ability due to its strong electron-absorbing reducing properties, capable of neutralizing all forms of ROS and inhibiting the accumulation of ROS-initiated lipid peroxidation [35,36]. Fullerol exhibited this powerful ability to scavenge ROS, also probably due to the non-localized double bonds on its molecular surface [37,38].

The antioxidant system is considered to be an important defense system for plants in response to environmental stress, preventing oxidative damage to cells caused by an excessive accumulation of ROS [39,40]. Antioxidant enzyme SOD can catalyze the disproportionation reaction of O_2_^−^ to produce H_2_O_2_ in plants, while CAT and POD are mainly responsible for decomposing and scavenging H_2_O_2_, and for further reducing ·OH accumulation [41,42]. Here, as well, the activities of SOD, CAT, and POD also increased in two wheat cultivars under drought stress. There was no significant difference in SOD enzymatic activity between the two cultivars of wheat, while the activities of CAT and POD in the CW131 cultivar were higher than they were in BM1. In agreement with this, Batool et al. [5] suggested that the drought-induced accumulation of antioxidant enzymes tended to be higher in modern cultivars than in old cultivars. Furthermore, exogenous fullerol treatment (25–100 mg L^−1^) promoted antioxidant enzyme activities compared to drought stress alone, particularly at 50 mg L^−1^. This was similar to the findings of previous research, which showed that the application of exogenous fullerol activated the antioxidant enzyme system in maize [18], sugar beets [13], canola [10], and wheat plants [25] under stressful conditions. In general, the improvements in antioxidant enzyme activities promoted by fullerol treatment under drought stress may be associated with the up-regulation of these antioxidant enzyme genes [7,35,43]. The mechanism of regulation of the antioxidant system by fullerol may also be mediated by ABA, resulting in the better adaptation of plants to dehydration [10]. Therefore, these results demonstrate that fullerol at specific concentrations could enhance the antioxidant system to alleviate cell membrane damage caused by ROS accumulation, thus allowing wheat plants to actively adapt to drought stress. In addition, there was no significant difference in the effect of fullerol on wheat between the two cultivars. Previous studies show that the regulation of plant growth by carbon nanomaterials is influenced by the concentration of fullerol, plant species and growth conditions [44,45].

## 4. Materials and Methods

Fullerol (purity > 99.9%) was provided by Chengdu Organic Chemicals Co. Ltd., Chinese Academy of Sciences (Chengdu, China). The experiment was carried out in a controlled plant growth chamber at Anhui Agriculture University, Hefei, China. The conditions of plant growth were controlled by the photoperiod for 14 h (07:00–21:00 h BST), under the day/night temperature of 25/20 °C, and an average humidity 85%. The two cultivars of winter wheat (*Triticum aestivum* L.), Bima 1 (BM1) and Changwu 131 (CW131), were selected for the experiment, which were obtained from Northwest Agriculture and Forestry University, Xi’an, China. BM1 was released in the 1950s, and CW131 was released in the 1990s. BM1 has many tillers and a weak drought resistance ability, while CW131 has large grain stalks, is of a high quality, and has a strong drought resistance ability. In this study, the seeds of two wheat cultivars of good quality and full and neat grain were selected and sterilized with 2% NaClO for 15 min, then washed 4–5 times with distilled water and reserved for the following experiments.

### 4.1. Trial 1: Effect of Fullerol on Seed Germination in Two Winter Wheat Cultivars

Trail 1 was carried out to reveal the influence of different concentrations of fullerol treatment on seed germination in two wheat cultivars under drought stress stimulated by PEG treatment.

#### 4.1.1. Characterization of Fullerol Nanoparticles

An atomic force microscope (AFM) (Multimode 8, Bruker, Billerica, MA, USA) was used to determine the dispersion and actual size of the synthesized fullerol nanoparticles. The fullerol nanoparticles were dissolved in H_2_O, freeze-dried, and characterized by Fourier transform infra-red (FTIR) (IN10-MX-IZ10, Thermo, Oxford, UK) using the potassium bromide tablet compression method. A thermogravimetric analysis (TGA) of fullerol nanoparticles was carried out using a thermogravimetric analyzer (STA 409 PC/PG, NETZSCH, Selb, Germany). The experimental conditions were as follows: the flow rate of nitrogen and air was 100 mL min^−1^; the temperature range was 30–800 °C; the heating rate was 10 °C min^−1^.

#### 4.1.2. Drought Stress and Fullerol Treatment, and Seed Germination Measurements

Fullerol was dissolved in a 20% PEG-6000 solution to prepare six stock concentrations (0, 25, 50, 100, 200 and 300 mg L^−1^), referred to as PEG + F_0_, PEG + F_25_, PEG + F_50_, PEG + F_100_, PEG + F_200_, and PEG + F_300_, respectively. Only distilled water served as a control. The fullerol concentrations were selected based on previous studies [10,12,18]. The seeds of two wheat cultivars were germinated under treatment with the different concentrations mentioned above. A layer of Whatman filter paper was placed in an 11 cm Petri dish, 10 mL of chemical solution was added, and 50 seeds were placed evenly on the filter paper. Each treatment consisted of three biological replicates. The germinated seeds were recorded every day for one week, and the radicle and germ length were measured on the 7th day. After that, the seed germination parameters were determined as follows:

Final germination percentage (%) = (n/N) × 100%, where n is the number of seeds that germinated, and N is the total number of seeds.

Germination index = Ʃ Gt/Dt, where Gt refers to the number of seeds germinated on day t and Dt is day 1, 2, 3, etc.

Seed vigor index = final gemination percentage (%) × [radical length + plumule length (cm)].

### 4.2. Trial 2: Effect of Fullerol on Drought Tolerance in Two Winter Wheat Cultivars

Trial 2 was carried out to investigate the influence of fullerol treatment on seedling growth, ROS levels, lipid peroxidation, and antioxidant enzyme activities in two cultivars of winter wheat under drought stress caused by PEG treatment.

#### 4.2.1. Plant Materials and Growth Conditions

The seeds of two wheat cultivars were germinated under six different concentrations of fullerol solution (0, 25, 50, 100, 200, and 300 mg L^−1^), for 7 days and then transferred to paper pots (9 cm in diameter and 9 cm in height) filled with 0.2 L of the Hoagland nutrient solution containing 20% PEG-6000, referred to as PEG + F_0_, PEG + F_25_, PEG + F_50_, PEG + F_100_, PEG + F_200_, PEG + F_300_, respectively. Only distilled water served as a control. There were seven treatments in this experiment. Each treatment consisted of three biological replicates. Each pot contained 13 wheat seedlings and was supplemented with 30 mL of a solution of different treatments every two days. The paper pots are wrapped in plastic bags to prevent water leakage. The leaf and root samples were harvested on the 10th day after PEG treatment.

#### 4.2.2. Measurement of Plant Height and Root Growth Parameters

Six wheat seedlings were randomly selected for each treatment, the plant height was measured, and the root system was carefully taken out and washed with distilled water. Roots were then scanned with a root morphology scanner (Epson Expression 12000XL Scanner, Seiko Epson Corp, Tokyo, Japan), and the root images were analyzed by the WinRHIZO system (Regent Instruments Inc., Québec, QC, Canada).

#### 4.2.3. Measurement of ROS Level, Malondialdehyde, and Antioxidant Enzyme Activities

An amount of 0.5 g of fresh leaves was ground to a powder using liquid N_2_, and then 5 mL of a pre-cooled 50 mM phosphate buffer (PBS) (pH 7.8) containing 1% polyvinyl pyrrolidone was added to be well-ground into a homogenate. The mixture was shaken well and placed at 4 °C overnight for full extraction, followed by centrifugation at 12,000× *g* 4 °C for 20 min [46]. The supernatant was taken, dispensed, and stored at −20 °C for the determination of the following indicators. ROS levels, lipid peroxidation, and antioxidant enzyme activities were all measured with tkits produced by Nanjing Jiancheng Bioengineering Institute (Nanjing, China).

Determination of ROS: The content of H_2_O_2_ was determined by measuring the absorbance of the complex formed by molybdic acid with H_2_O_2_ at 405 nm using a H_2_O_2_ assay kit. The content of H_2_O_2_ was expressed in mmol g^−1^ protein. The hydroxyl radical (·OH) was assayed by monitoring the absorbance of the red substance at 550 nm formed by the Griess reagent in the Fenton reaction using a ·OH assay kit. The ·OH content was expressed in units (U) mg^−1^ protein.

Lipid peroxidation: Lipid peroxidation was measured as the content of malondialdehyde (MDA) by monitoring the absorbance of the red complex at 532 nm produced by the condensation of thiobarbituric acid (TBA) with MDA in accordance with the method of the MDA assay kit. The MDA content was expressed in nmol mg^−1^ protein.

Antioxidant enzyme activity assay: The SOD activity was determined by monitoring the inhibition rate of the enzyme on the production of superoxide by the xanthine and xanthine oxidase reaction system using a SOD assay kit. The CAT activity was calculated by the decrease in the absorbance of H_2_O_2_ at 405 nm using CAT assay kit. The POD activity was assayed based on the absorbance change at 420 nm by catalyzing H_2_O_2_. Antioxidant enzyme activities were all expressed in units (U) mg^−1^ protein.

### 4.3. Statistical Analysis

All the data were examined by two-way ANOVA to test the main effects (of fullerol and cultivar treatment) and their interactions using SPSS software version 17.0 (IBM SPSS Inc., Chicago, IL, USA). The means were separated using a LSD test at the 5% level of significance. The linear, quadratic and one-way ANOVA terms for fullerol addition were used in a lack-of-fit test, aiming to test which model was best supported [47].

## 5. Conclusions

This study demonstrated that seeds exposed to fullerol at appropriate concentrations (25–200 mg L^−1^) significantly increased seed germination in two wheat cultivars under drought stress; the most significant effective concentration was 50 mg L^−1^. We also confirmed that seed priming with 50 and 100 mg L^−1^ of fullerol was more pronounced in promoting seedling growth and antioxidant enzyme activities (SOD, CAT, and POD), as well as in reducing ROS and MDA accumulation in both wheat cultivars under drought stress. In addition, modern cultivars (CW131) showed better drought adaptation than old cultivars (BM1) did, which is mainly due to the genotype. Moreover, the effect of fullerol on wheat showed no significant difference between the two cultivars. More importantly, it is suggested that fullerol was an efficient free radical scavenger and could be an excellent candidate for stimulating plant defense under stressful conditions. Therefore, it can be concluded that exogenous fullerol ameliorated the inhibition effects of drought on wheat and promoted seed germination, early seedling growth and adaptation under drought stress. In the future, it is essential to evaluate the long-term effects of fullerol on different crops and to estimate appropriate concentrations of it for regulating crop growth under stressful conditions.

## Figures and Tables

**Table 1:** Effect of different fullerol additions (concentrations range from 0 to 300 mg L^−1^) on the seed germination parameters of two wheat cultivars (CW131 and BM1) under PEG (20% polyethylene glycol) treatment.

Cultivar	Treatment	Final Germination Percentage (%)	Germination Index	Radical Plus Plumule Length (cm)	Seed Vigor Index
CW131	Control	98.7 ± 0.7 ^a^	44.0 ± 1.4 ^a^	15.7 ± 0.1 ^a^	1545.1 ± 13.0 ^a^
	PEG + F_0_	87.3 ± 3.7 ^c^	20.3 ± 1.5 ^d^	5.3 ± 0.1 ^d^	460.5 ± 19.9 ^e^
	PEG + F_25_	94.7 ± 1.3 ^ab^	25.1 ± 0.6 ^bc^	6.3 ± 0.1 ^c^	599.4 ± 9.0 ^c^
	PEG + F_50_	99.3 ± 0.7 ^a^	27.7 ± 1.8 ^b^	7.1 ± 0.1 ^b^	710.0 ± 9.9 ^b^
	PEG + F_100_	96.0 ± 0.0 ^ab^	23.3 ± 0.1 ^c^	6.2 ± 0.2 ^c^	595.2 ± 15.5 ^c^
	PEG + F_200_	96.7 ± 0.7 ^a^	23.4 ± 0.2 ^c^	5.5 ± 0.2 ^d^	536.3 ± 18.3 ^d^
	PEG + F_300_	91.3 ± 0.7 ^bc^	21.3 ± 0.3 ^d^	5.5 ± 0.3 ^d^	506.9 ± 26.7 ^de^
BM1	Control	99.3 ± 0.7 ^a^	40.7 ± 0.8 ^a^	13.2 ± 0.1 ^a^	1313.1 ± 6.1 ^a^
	PEG + F_0_	82.7 ± 3.5 ^c^	19.3 ± 1.4 ^d^	3.6 ± 0.2 ^d^	301.4 ± 25.0 ^d^
	PEG + F_25_	89.3 ± 1.8 ^b^	21.6 ± 0.3 ^bc^	4.8 ± 0.5 ^c^	427.0 ± 7.4 ^c^
	PEG + F_50_	90.7 ± 2.9 ^b^	23.3 ± 0.5 ^b^	6.8 ± 0.3 ^b^	620.5 ± 46.3 ^b^
	PEG + F_100_	90.0 ± 2.0 ^b^	21.3 ± 0.6 ^bc^	5.2 ± 0.4 ^c^	468.1 ± 37.1 ^c^
	PEG + F_200_	87.3 ± 1.3 ^bc^	20.5 ± 0.3 ^cd^	5.1 ± 0.2 ^c^	447.7 ± 15.2 ^c^
	PEG + F_300_	87.0 ± 0.6 ^bc^	20.1 ± 0.4 ^cd^	3.9 ± 0.3 ^d^	339.0 ± 24.1 ^d^
	Cultivar (C)	**	**	**	**
	Fullerol (F)	**	**	**	**
	C × F	ns	ns	*	ns

Mean values ± SE are shown (*n* = 3). Values with the same letter in the same column for one cultivar are not significantly different at *p* < 0.05. For ANOVA results, *, *p* < 0.05; **, *p* < 0.01; ns, not significant.

**Table 2:** Effect of different fullerol additions (concentrations range from 0 to 300 mg L^−1^) on the root growth parameters of two winter cultivars (CW131 and BM1) under PEG (20% polyethylene glycol) treatment.

Cultivar	Treatment	Root Length (cm)	Root Surface Area (cm^2^)	Root Volume (cm^3^)	Root Mean Diameter (mm)
CW131	Control	53.9 ± 2.3 ^a^	6.4 ± 0.1 ^a^	0.055 ± 0.001 ^a^	0.37 ± 0.02 ^ab^
	PEG + F_0_	40.4 ± 1.3 ^cd^	4.1 ± 0.1 ^d^	0.036 ± 0.001 ^c^	0.37 ± 0.01 ^ab^
	PEG + F_25_	44.8 ± 1.7 ^bc^	4.6 ± 0.1 ^cd^	0.036 ± 0.002 ^c^	0.37 ± 0.02 ^ab^
	PEG + F_50_	47.8 ± 0.1 ^b^	5.2 ± 0.2 ^b^	0.046 ± 0.001 ^b^	0.39 ± 0.01 ^a^
	PEG + F_100_	44.4 ± 1.8 ^bc^	4.8 ± 0.4 ^bc^	0.044 ± 0.004 ^b^	0.37 ± 0.01 ^ab^
	PEG + F_200_	42.3 ± 1.4 ^cd^	4.6 ± 0.1 ^cd^	0.041 ± 0.005 ^bc^	0.36 ± 0.01 ^ab^
	PEG + F_300_	39.6 ± 0.5 ^d^	4.4 ± 0.3 ^cd^	0.034 ± 0.002 ^c^	0.35 ± 0.05 ^b^
BM1	Control	50.8 ± 0.6 ^a^	6.1 ± 0.4 ^a^	0.052 ± 0.005 ^a^	0.34 ± 0.03 ^a^
	PEG + F_0_	38.5 ± 0.8 ^c^	4.0 ± 0.1 ^cd^	0.034 ± 0.006 ^c^	0.33 ± 0.01 ^a^
	PEG + F_25_	39.3 ± 1.3 ^c^	4.5 ± 0.2 ^bc^	0.038 ± 0.006 ^bc^	0.36 ± 0.02 ^a^
	PEG + F_50_	44.9 ± 1.0 ^b^	4.9 ± 0.2 ^b^	0.044 ± 0.001 ^ab^	0.37 ± 0.01 ^a^
	PEG + F_100_	40.7 ± 0.6 ^c^	4.7 ± 0.1 ^bc^	0.036 ± 0.002 ^bc^	0.35 ± 0.02 ^a^
	PEG + F_200_	38.2 ± 1.7 ^c^	4.2 ± 0.3 ^bcd^	0.033 ± 0.003 ^c^	0.34 ± 0.02 ^a^
	PEG + F_300_	37.5 ± 1.5 ^c^	3.8 ± 0.3 ^d^	0.031 ± 0.002 ^c^	0.33 ± 0.02 ^a^
	Cultivar (C)	**	*	*	*
	Fullerol (F)	**	**	**	ns
	C × F	ns	ns	ns	ns

Mean values ± SE are shown (*n* = 3). Values with the same letter in the same column for one cultivar are not significantly different at *p* < 0.05. For ANOVA results, *, *p* < 0.05; **, *p* < 0.01; ns, not significant.

## Data Availability

The data in this study are available from the corresponding author upon request.

## Supplementary Materials

The following supporting information can be downloaded at https://www.mdpi.com/article/10.3390/plants12061417/s1, Figure S1: TG-GTA of fullerol; Table S1: Elemental analysis of fullerol; Figure S2: Quadratic polynomial regression; Table S2–S7: Comparison of linear model, quadratic model and one-way ANOVA.
