# Supplementary material for: Seed Priming with Fullerol Improves Seed Germination, Seedling Growth and Antioxidant Enzyme System of Two Winter Wheat Cultivars under Drought Stress
Source: Plants (Basel). 2023 Mar 22;12(6):1417. doi: 10.3390/plants12061417 (PMC10052877; doi:10.3390/plants12061417)
Supplement: Supplementary file 1 [file plants-12-01417-s001.zip › plants-2260500-supplementary.pdf]

supplementary materials

Table S1 Elemental analysis of fullerol

Weight (mg)	Number	N Area	C Area	H Area	S Area	N (%)	C (%)	H (%)	S (%)
1.6660	1	0	19 038	3 829	53	0.00	37.97	2.453	0.186

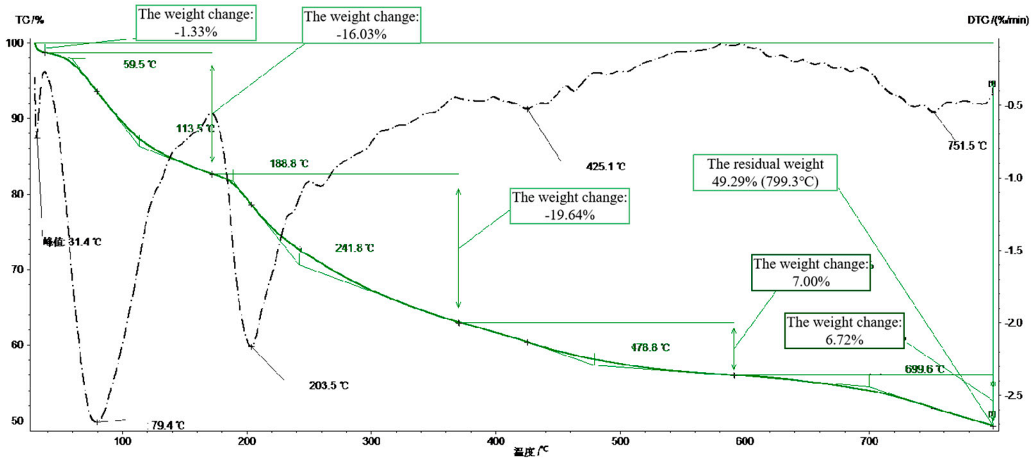

Figure S1. TG-GTA of fullerol

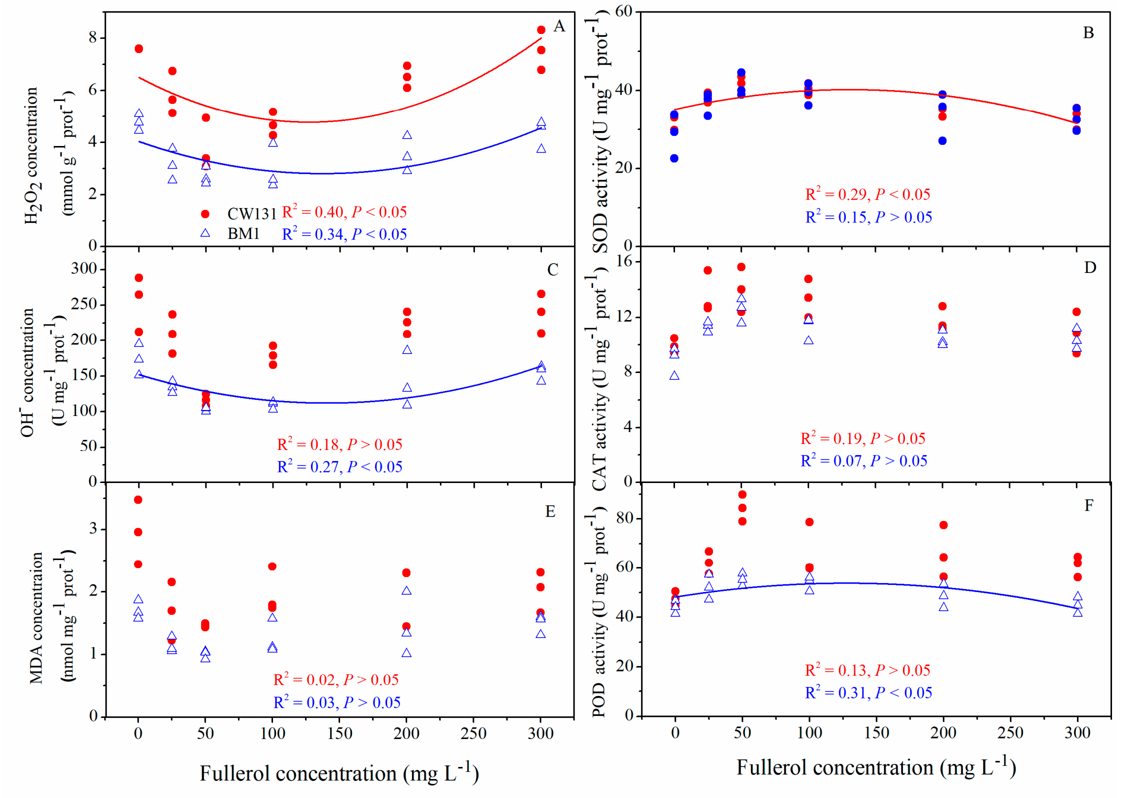

Figure S2. Contents of hydrogen peroxide (H_2O_2) (A), hydroxyl radical (·OH) (C), malondialdehyde (MDA) (E), and Activities of antioxidant enzymes: superoxide dismutase (SOD) (B), catalase (CAT) (D), and pe-roxidase (POD) (F) of two winter cultivars (CW131 and BM1) plotted against six concentrations of fullerol (0, 20, 50, 100, 200, 300 mg L^{-1}).

Table S2. The result comparing the regression curve of the response of hydrogen peroxide (H_2O_2) to fullerol addition. These regressions were done using linear model, quadratic model, and One-way ANOVA model via lack of fit test function of the library of R.

	**Source**	***df* of regression**	***df* of error**	***F* value**	***P***	***R*²**
**CW131**	**Linear** (x as contious variable)	1	16	2.090	0.168	0.060
	**One-way ANOVA** (x as discrete variable)	5	12	15.980	< 0.001	0.815
	**Quadratic** (x as contious variable)	2	15	6.726	0.008	0.403
	**lack of fit between Linear and Quadratic**	−1		10.166	0.006	
	**lack of fit between One-way ANOVA and Quadratic**	3		12.149	< 0.001	
**BM1**	**Linear** (x as contious variable)	1	16	0.624	0.441	−0.023
	**One-way ANOVA** (x as discrete variable)	5	12	5.825	0.006	0.587
	**Quadratic** (x as contious variable)	2	15	5.336	0.018	0.338
	**lack of fit between Linear and Quadratic**	−1		9.709	0.007	

	**lack of fit between One-way ANOVA and Quadratic**	3		4.009	0.034	

Table S3. The results comparing the regression curve of the response of hydroxyl radical (·OH) to fullerol addition. These regressions were done using linear model, quadratic model, and One-way ANOVA model via lack of fit test function of the library of R.

	**Source**	***df* of regression**	***df* of error**	***F* value**	***P***	***R^2***
**CW131**	**Linear** (x as contious variable)	1	16	1.083	0.314	0.005
	**One-way ANOVA** (x as discrete variable)	5	12	12.720	< 0.001	0.775
	**Quadratic** (x as contious variable)	2	15	2.844	0.090	0.178
	**lack of fit between Linear and Quadratic**	−1		4.376	0.054	
	**lack of fit between One-way ANOVA and Quadratic**	3		14.270	< 0.001	
**BM1**	**Linear** (x as contious variable)	1	16	0.292	0.596	-0.043
	**One-way ANOVA** (x as discrete variable)	5	12	5.651	0.007	0.578
	**Quadratic** (x as contious variable)	2	15	4.211	0.035	0.274
	**lack of fit between Linear and Quadratic**	−1		8.002	0.013	
	**lack of fit between One-way ANOVA and Quadratic**	3		4.593	0.023	

Table S4. The results comparing the regression curve of the response of malondialdehyde (MDA) to fullerol addition. These regressions were done using linear model, quadratic model, and One-way ANOVA model via lack of fit test function of the library of R.

	**Source**	***df* of regression**	***df* of error**	***F* value**	***P***	***R^2***
**CW131**	**Linear** (x as contious variable)	1	16	0.225	0.642	−0.048
	**One-way ANOVA** (x as discrete variable)	5	12	4.800	0.012	0.528
	**Quadratic** (x as contious variable)	2	15	1.214	0.325	0.025
	**lack of fit between Linear and Quadratic**	−1		2.186	0.160	
	**lack of fit between One-way ANOVA and Quadratic**	3		6.328	0.008	
**BM1**	**Linear** (x as contious variable)	1	16	0.546	0.471	−0.027
	**One-way ANOVA** (x as discrete variable)	5	12	2.974	0.057	0.367
	**Quadratic** (x as contious variable)	2	15	1.243	0.317	0.028
	**lack of fit between Linear and Quadratic**	−1		1.909	0.187	
	**lack of fit between One-way ANOVA and Quadratic**	3		3.683	0.043	

Table S5. The results comparing the regression curve of the response of superoxide dismutase (SOD) to fullerol addition. These regressions were done using linear model, quadratic model, and One-way ANOVA model via lack of fit test function of the library of R.

	Source	*df* of regreession	*df* of error	*F* value	*P*	R^2
**CW131**	**Linear** (x as contious variable)	1	16	1.191	0.291	0.011
	**One-way ANOVA** (x as discrete variable)	5	12	20.050	< 0.001	0.849
	**Quadratic** (x as contious variable)	2	15	4.408	0.031	0.286
	**lack of fit between Linear and Quadratic**	−1		7.166	0.017	
	**lack of fit between One-way ANOVA and Quadratic**	3		19.568	< 0.001	
**BM1**	**Linear** (x as contious variable)	1	16	0.252	0.623	−0.046
	**One-way ANOVA** (x as discrete variable)	5	12	3.707	0.029	0.443
	**Quadratic** (x as contious variable)	2	15	2.521	0.114	0.152
	**lack of fit between Linear and Quadratic**	−1		4.732	0.046	
	**lack of fit between One-way ANOVA and Quadratic**	3		3.618	0.046	

Table S6. The results comparing the regression curve of the response of catalase (CAT) to fullerol addition. These regressions were done using linear model, quadratic model, and One-way ANOVA model via lack of fit test function of the library of R.

	Source	*df* of regreession	*df* of error	*F* value	*P*	R^2
**CW131**	**Linear** (x as contious variable)	1	16	0.953	0.344	−0.003
	**One-way ANOVA** (x as discrete variable)	5	12	4.887	0.011	0.533
	**Quadratic** (x as contious variable)	2	15	3.053	0.077	0.195
	**lack of fit between Linear and Quadratic**	−1		4.920	0.042	
	**lack of fit between One-way ANOVA and Quadratic**	3		4.631	0.023	
**BM1**	**Linear** (x as contious variable)	1	16	0.063	0.805	−0.058
	**One-way ANOVA** (x as discrete variable)	5	12	7.502	0.002	0.657
	**Quadratic** (x as contious variable)	2	15	1.627	0.229	0.069
	**lack of fit between Linear and Quadratic**	−1		3.182	0.095	
	**lack of fit between One-way ANOVA and Quadratic**	3		9.562	0.002	

Table S7. The results comparing the regression curve of the response of peroxidase (POD) to fullerol addition. These regressions were done using linear model, quadratic model, and One-way ANOVA model via lack of fit test function of the library of R.

	Source	*df* of regreession	*df* of error	*F* value	*P*	R^2
**CW131**	**Linear** (x as contious variable)	1	16	0.025	0.876	−0.061
	**One-way ANOVA** (x as discrete variable)	5	12	8.299	0.001	0.682
	**Quadratic** (x as contious variable)	2	15	2.287	0.136	0.132
	**lack of fit between Linear and Quadratic**	−1		4.544	0.050	
	**lack of fit between One-way ANOVA and Quadratic**	3		9.665	0.002	
**BM1**	**Linear** (x as contious variable)	1	16	1.591	0.225	0.034
	**One-way ANOVA** (x as discrete variable)	5	12	4.782	0.012	0.527
	**Quadratic** (x as contious variable)	2	15	4.778	0.025	0.308
	**lack of fit between Linear and Quadratic**	−1		7.335	0.016	
	**lack of fit between One-way ANOVA and Quadratic**	3		3.312	0.057	
